# Effects of Negative Pressure Wound Therapy on Mesenchymal Stem Cells Proliferation and Osteogenic Differentiation in a Fibrin Matrix

**DOI:** 10.1371/journal.pone.0107339

**Published:** 2014-09-12

**Authors:** Jin Zhu, Aixi Yu, Baiwen Qi, Zonghuan Li, Xiang Hu

**Affiliations:** Department of Micro-Orthopedics, Zhongnan Hospital of Wuhan University, Wuhan, China; Department of Biomaterials, Japan

## Abstract

Vacuum-assisted closure (VAC) negative pressure wound therapy (NPWT) has been proven to be an effective therapeutic method for the treatment of recalcitrant wounds. However, its role in bone healing remains to be unclear. Here, we investigated the effects of NPWT on rat periosteum-derived mesenchymal stem cells (P-MSCs) proliferation and osteoblastic differentiation in a 3D fibrin matrix. P-MSCs underwent primary culture for three passages before being used to construct cell clots. The fibrin clots were incubated with NPWT under continuous suction at −125 mmHg in a subatmospheric perfusion bioreactor. Clots exposed to atmospheric pressure served as the static control. Compared to the control group, cell proliferation significantly increased in NPWT group after incubation for 3 days. There was no statistical difference in apoptosis rate between two groups. The ALP activity and mineralization of P-MSCs all increased under continuous suction. The expressions of collagen type 1 and transcription factor Cbfa-1 were higher at the 1-, 3-, and 7-day timepoints and the expressions of osteocalcin and integrin β5 were higher at the 3-, and 7-day timepoints in the NPWT group. These results indicate that a short time treatment with NPWT, applied with continuous suction at −125 mmHg, can enhance cellular proliferation of P-MSCs and induce the differentiation toward an osteogenic phenotype. The mechanotransduction molecule integrin β5 was found to be highly expressed after NPWT treatment, which indicates that NPWT may play a positive role in fracture healing through enhance bone formation and decrease bone resorption.

## Introduction

Negative pressure wound therapy (NPWT) has been proven to be effective at treating complex wounds [1]–[4]. This therapy can promote granulation tissue formation and improve wound healing through reducing tissue edema, removing factors that inhibit wound healing, increasing wound perfusion, and reducing incidence of infection [5]–[8]. However, controversy still remains regarding the benefits of NPWT on bone tissue, though there have been many clinic and basement reports about its application in treatment of open fracture or osseous defect combined with soft tissue injury, and these studies nearly all show that NPWT can effectively improve wound and bone healing or has no deleterious effect on fracture healing [9]–[12].

Bone regeneration is a complex process involving many cells and factors from different compartments of the bone. Mesenchymal stem cells (MSCs) from periosteum, endosteum and bone marrow play a pivotal role in bone healing. They may contribute to healing through osteogenic and chondrogenic differentiation, endochondral ossification, and/or through the release of paracine factors resulting in recruitment and activation of host osteoprogenitor cells [13]. Except for the above factors, mechanical strain is also one important factor in the regulation of bone modeling and remodeling [14]. Yamazaki et al [15] showed that a lack of mechanical stress can significantly reduce the capability of MSCs to differentiate into an osteoblast. Numerous in vitro researches have also demonstrated that mechanical stimuli and hydrostatic pressure can enhance cellular viability and improve osteogenic differentiation and maturation of MSCs [16]–[20]. Swain et al [21] reported NPWT activates within mature dura a natural healing cascade that results in osseous tissue formation using a rabbit cranial critical-size defects model, they reasoned that negative pressure-induced mechanical signals (tissue stretching) may promote the process that progenitor cells from the dura differentiate to osteoblasts and then synthesize bone matrix with subsequent mineralization. Therefore, we hypothesize that the application of NPWT in traumatic wound with fracture or segmental bone loss may result in the transduction of strain to the underlying periosteum, with concomitant cell stretching, stimulating osseous healing in an analogous manner.

Wilkes et al [22] firstly described an in vitro NPWT system. They developed a fibrin matrix to support cellular growth, withstand the suction forces generated during subatmospheric pressure application, and allow culture medium flow through the matrix. This new culture system mimics the wound micro-environment and permits the study of the cellular changes under NPWT in vitro. In this study, to show the correlation between NPWT and osteogenic differentiation of periosteum-derived mesenchymal stem cells (P-MSCs), we assembled a bioreactor which is similar to the above report, and then investigated the effects of NPWT on P-MSCs that were the initial stage in the process of osteogenic differentiation by induction with dexamethasone, ascorbic acid and β-glycerophosphate. As this experiment here is the first to explore the effects of NPWT on P-MSCs, we chose the subatmospheric value −125 mmHg, which is most effective for soft-tissue wounds [2], [5], [23]. We examined cellular viability, alkaline phosphatase (ALP) activity, mineralization and the expression levels of ostoblast-related genes and proteins under NPWT or static control. The aim of this work was to detect the possible role of NPWT in bone healing.

## Materials and Methods

### Ethics statement

All experiments were approved by the Institutional Animal Care and Use Committee of Wuhan University, and the animal procedures were performed in strict accordance with institutional and national guidelines. All efforts were made to minimize suffering.

### Cell isolation and culture

Twelve 4-week-old male Sprague-Dawley rats were sacrificed for the present study. P-MSCs were isolated and characterised in accordance with published techniques [24]. Briefly, the rats were sacrificed by cervical dislocation and sterilized in 75% ethanol for 5 min before surgery. The periosteum was stripped off mechanically from femora and tibiae using forceps, then minced, digested for 2 h at 37°C with 0.2% type II collagenase (Sigma-Aldrich, St.Louis, MO, USA). The released cells were collected by centrifugation (1,000 rpm for 5 minutes) after filtration by 40-µm cell strainers, and maintained in expansion medium consisting of L-DMEM (Hyclone) and 10% FBS (Gibco) supplemented with 2 mM L-glutamine, 100 U/ml penicillin and 100 U/ml streptomycin in a humidified atmosphere containing 5% CO_2_ at 37°C. For subculture, cells were detached with 0.25% trypsin (Amresco) and passaged at a ratio of 1∶2 plates when cells grew to 70–80% confluence. P-MSCs were identified as CD44(+), CD90(+), and CD34(−); furthermore, these cells cultured in adipogenic or osteogenic media differentiated into adipocytes and osteoblasts, respectively (Figure S1). P-MSCs were used for experiments from the second to third passages.

### Fibrin matrix and cell clots preparation

Fibrin clots were prepared as described [22]. Briefly, porcine plasma (Lampire Biological Laboratories Inc, Pipersville, PA) supplemented with fibrinogen (Sigma) to a final fibrinogen concentration of 9.8 mg/ml was prepared. To form the bottom layer, 600 µl porcine plasma was added to each insert (BD Falcon: 6-well format, 1 µm pore size) and then was clotted by 150 µl of thrombin solution (1800 U/ml). The top layer was formed by adding 600 µl porcine plasma with 20,000 P-MSCs on the top of first clot layer. Another 150 µl of thrombin was then added to encapsulate the cells. Double-layered clots were incubated at 37°C for 1 hour before adding cell culture medium.

### NPWT treatment using a bioreactor

We assembled the bioreactor modifying from the previous report [22]. Briefly, the inserts were placed through one O-sealing ring and then into the well of a 6-well plate. A layer of foam (VSD Inc, Wuhan, China) was placed on the top of the clot. To create a seal, a drape was placed on the top of the wells. One needle accessed through a 3 M bumpon was inserted into the foam and connected to a vacuum pump (VSD Inc.) that generated continuously suction at −125 mmHg. Another needle with an adhesive-backed elastomeric disc was placed in the O-ring and reached the bottom of the plate. This needle was used to inject media through a peristaltic pump (Longer, Baoding, China) at 8 ml per 24 hours per well. As a comparator, control inserts were cultured in the same bioreactor without suction. For osteogenic differentiation assays, all cell clots were initially maintained with osteogenic medium (serum-containing DMEM supplemented with 50 mg/ml ascorbic acid, 10 mM β-glycerophosphate, and 0.1 mM dexamethasone [all from Sigma]) at 37°C and 5% CO_2_ for 7 days, and then were cultured in the same medium with or without NPWT for another 7 days. As for other experiments, constructs were evaluated after 3 days of incubation using expansion medium. All experiments were repeated 3 times in each of the following assays.

### Cell counting kit-8 assay

Following 72 hours of treatment with NPWT or static conditions, the cell clots were fed with fresh media containing a water soluble tetrazolium salt from the cell counting kit-8 (CCK-8, Dojindo, Japan). The experiment was carried out according to the manufacturer’s protocol. The absorbance at 450 nm was measured on a microplate reader.

### TUNEL Assay

Following 72 hours of treatment with NPWT or static conditions, the cell clots were fixed with 4% paraformaldehyde, and then were permeabilized in 0.5% Triton X-100, followed by freshly prepared TUNEL reaction mixture for 1 h in a dark room. The nuclei were stained by DAPI in mounting medium. Cells in clots were visualized and photographed using a confocal microscope (LSM710, ZEISS, Germany). Percent apoptosis was determined by counting the number of TUNEL^+^ cells and dividing by the total number of cells in randomly selected areas.

### ALP staining and activity assay

Following treatment with or without NPWT for 3 and 7 days, ALP staining was performed using leukocyte alkaline phosphatase (LAP) kits (Sigma) according to the manufacturers’ instruction. ALP activity was determined by measuring the OD values for absorbance at 405 nm after incubation with p-nitrophenyl phosphate (Sigma) for 15 min. The calibration curve was derived from a standard dilution series of p-nitrophenol (pNP, Sigma) which was prepared with concentrations ranging from 0 to 0.2 µmol/ml. ALP activity was expressed as nanomoles of pNP/µg DNA×min.

### Matrix Mineralization

Alizarin red staining was used to characterize mineralization of the cells. Following treatment with or without NPWT for 3 and 7 days, the clots were incubated with 2% Alizarin red S (Sigma) for 30 min. After that, they were washed with PBS and imaged using an Olympus Inverted Microscope. Following imaging, the stain was eluted off the cells with 10% cetylpyridinium chloride in 10 mM sodium phosphate solution for 15 min at room temperature. The absorbance was then measured at 590 nm using a spectrophotometer.

### Immunofluorescence staining

Following treatment with or without NPWT for 3 and 7 days, the cell clots were collected and fixed in 4% paraformaldehyde and permeabilized with 0.5% Triton X-100 at room temperature. Then they were blocked with goat serum, and incubated with the specific rat primary antibodies against collagen type 1 (COL1) (1∶100, BOSTER, Wuhan, China) overnight at 4°C. The clots were then incubated with a red fluorescently labeled goat anti-mouse secondary antibody for another 30 minutes at 37°C. The nuclei were stained with DAPI. After washing three times with PBS, the clots were viewed under a confocal laser-scanning microscope.

### Real-time PCR analysis

Following treatment with or without NPWT for 3 and 7 days, total RNA was extracted using Trizol (Invitrogen). Reverse transcription was carried out using 1 µg total RNA in final volume of 20 µL with PrimeScript RT reagent kit (Takara Bio, Shiga, Japan) according to the manufacturer’s recommendations. Real-time PCR was performed in an ABI 7900HT system using a real-time PCR kit (Takara Bio). The settings were as follows: denaturation at 95°C for 10 sec, and then 40 cycles (10 sec at 95°C, 30 sec at 60°C). A dissociation curve was constructed using the system software program v2.1 to confirm that there was no non-specific amplification. The housekeeping gene β-actin was used to be endogenous control for normalization. The primer sequences used in this study were listed in Table 1.

**Table 1:** Primer sequences for real-time RT-PCR.

Genes	Forward primer (5′-3′)	Reverse primer (5′-3′)
*Alpl*	TGGACGGTGAACGGGAGAAC	GCCATGACGTGGGGGATGTA
*Bglap*	CAAGTCCCACACAGCAACTC	TCACCACCTTACTGCCCTCC
*Cbfa1/Runx2*	GCTTCTCCAACCCACGAATG	GAACTGATAGGACGCTGACGA
*Col1a1*	CAGGCTGGTGTGATGGGATT	CCAAGGCATTCTGCCTCT
*Itgb5*	CGAGCTTGGGATAAAGCAAG	TCAACAGGCATCTCAACAGC
*Actb*	CGTTGACATCCGTAAAGACCTC	TAGGAGCCAGGGCAGTAATCT

### Western-blot analysis

Following treatment with or without NPWT for 3 and 7 days, the cells in fibrin clots were lysed using the protein extractiong reagent RIPA (Beyotime, Shanghai, China) containing 1 mM phenylmethylsulfonyl fluoride (Sigma) and a phosphatase-inhibitor cocktail (Sigma). Samples (50 ng protein) were separated by SDS polyacrylamide gel electrophoresis and electrotransferred onto Hybond-polyvinylidene fluoride (PVDF) membrane. The membranes were then incubated with rabbit anti-RUNX2 antibody (1∶200) (Santa Cruz, Inc.), rabbit anti-osteocalcin (OC) antibody (1∶400) (Abcam, Inc.), rabbit anti-integrin β5 (ITGB5) antibody (1∶1000) (Santa Cruz, Inc.) or rabbit anti-β-actin antibody (1∶1000) (BOSTER) in TBST overnight at 4°C. Horseradish peroxidase conjugated goat anti-rabbit IgG Ab (1∶1000) (Santa Cruz, Inc.) were used as secondary antibody. The protein bands were detected by ECL chemiluminescence. The results were quantified by Image J software.

### Statistical analysis

In all experiments, data were expressed as mean ± standard. Statistical comparisons were performed using Student’s *t*-test which was carried out using SPSS, v.18.0. *P-*value<0.05 was considered statistically significant.

## Results

### Cell morphology

**Figure 1:**
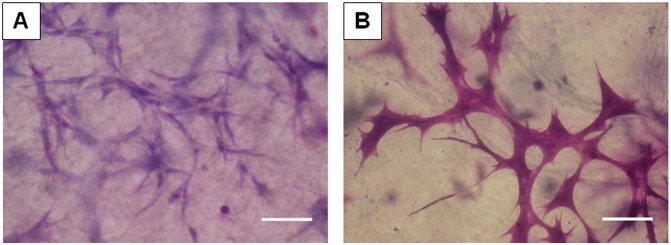
Effects of NPWT on morphological change. Control cells were thin with elongated spindle shape (A). The cells that were applied with NPWT were larger and thicker with many dendrite-like branches (B). Scale bar represents 100 µm.

Visual inspection of the cells using Wright–Giemsa staining revealed morphological differences between the two groups. The cells in Figure 1A are representative of static P-MSCs, thin with elongated spindle shape. Compared with control group, the cells in NPWT group appeared larger and thicker with dendrite-like branching and spread more (Figure 1B).

### Cell proliferation

**Figure 2:**
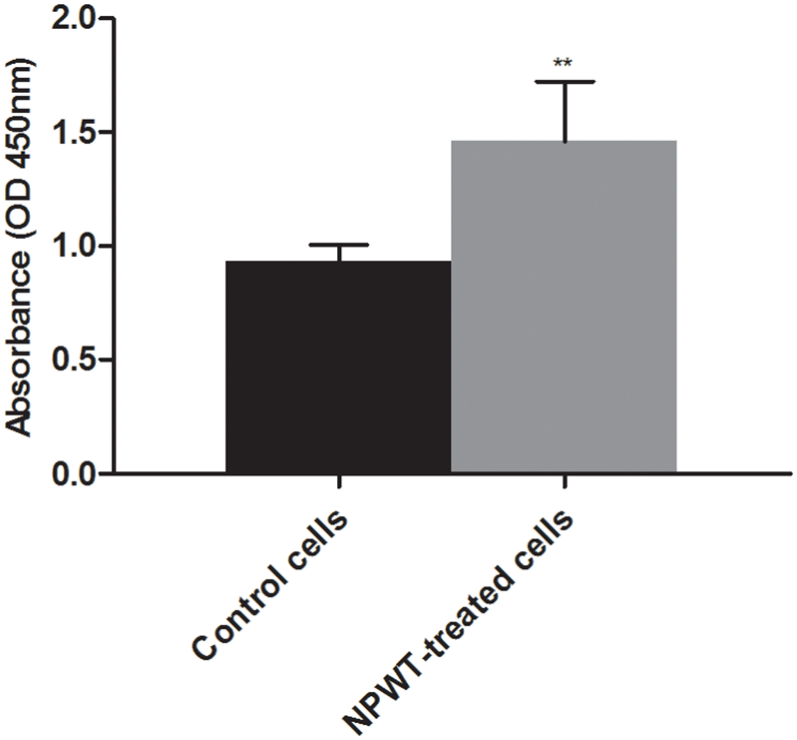
Cell proliferation assay. Following 72 hours of treatment with suction or static conditions, clots were exposed to water soluble tetrazolium salt-8-containing medium for 4 hours and optical density values at 450 nm were measured. Compared with control cells, NPWT-treated cells had a higher OD value (**p<0.01).

**Figure 3:**
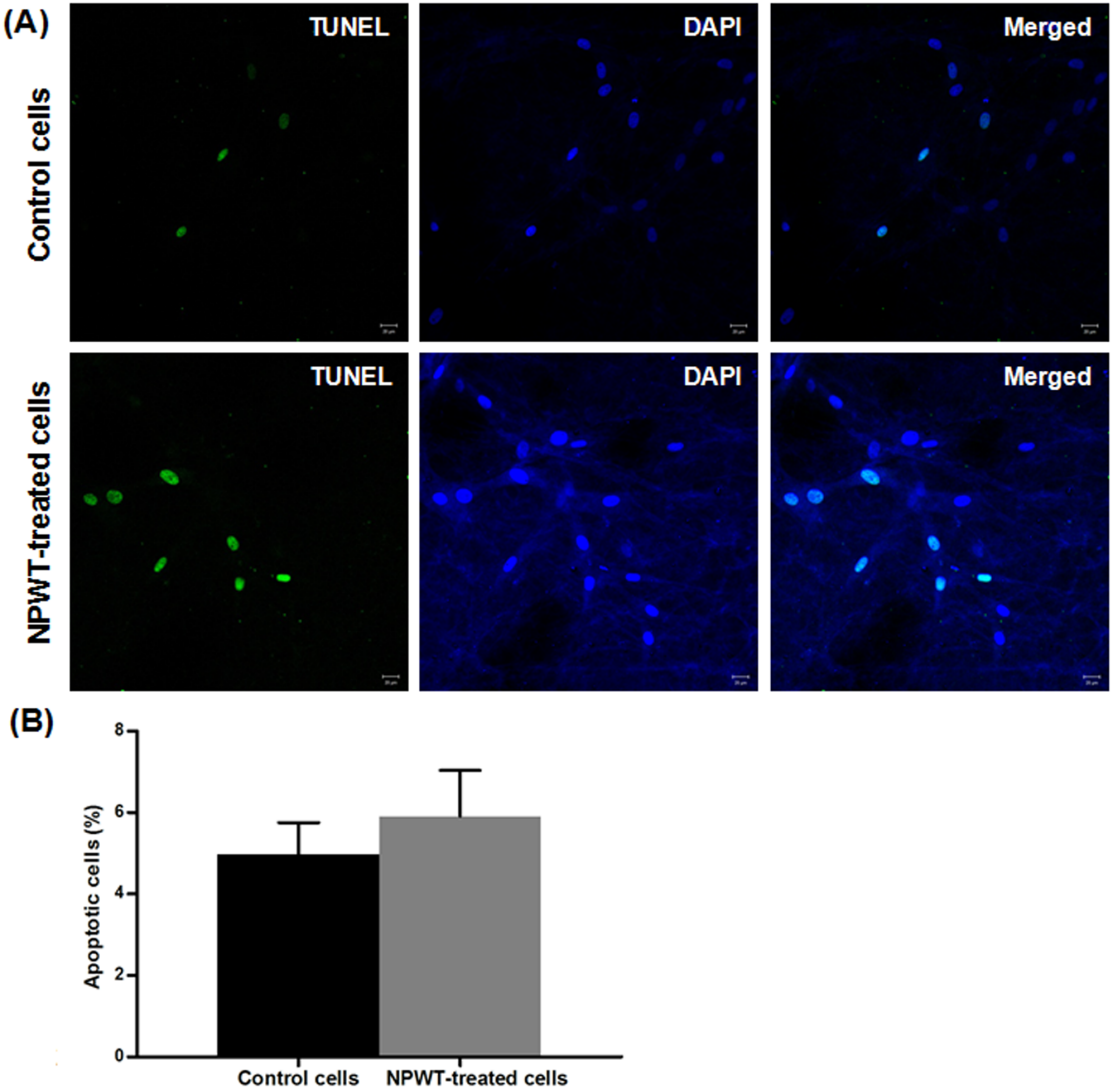
Cell apoptosis assay. Following 72 hours of treatment with suction or static conditions, apoptosis of MSCs was evaluated by TUNEL assay. The images of TUNEL^+^ cells was shown in (A, bars = 20 µm). Percentages of apoptotic cells in clots are shown in (B). Compared with control group, 72 hours of suction didn’t result in a significant increase in cell apoptosis (*p*>0.05).

The CCK-8 assay showed that the proliferation of P-MSCs in NPWT group was significantly upregulated compared to the control (Figure 2). We also performed TUNEL assay to observe if NPWT induced P-MSCs apoptosis. As shown in Figure 3A, treatment with continuous suction at −125 mmHg for 72 hours didn’t dramatically increase the number of TUNEL^+^ cells in P-MSCs. There was no significant difference in the percentage of apoptosis cells between the NPWT group and control group (Figure 3B).

### Osteogenic differentiation of P-MSCs

**Figure 4:**
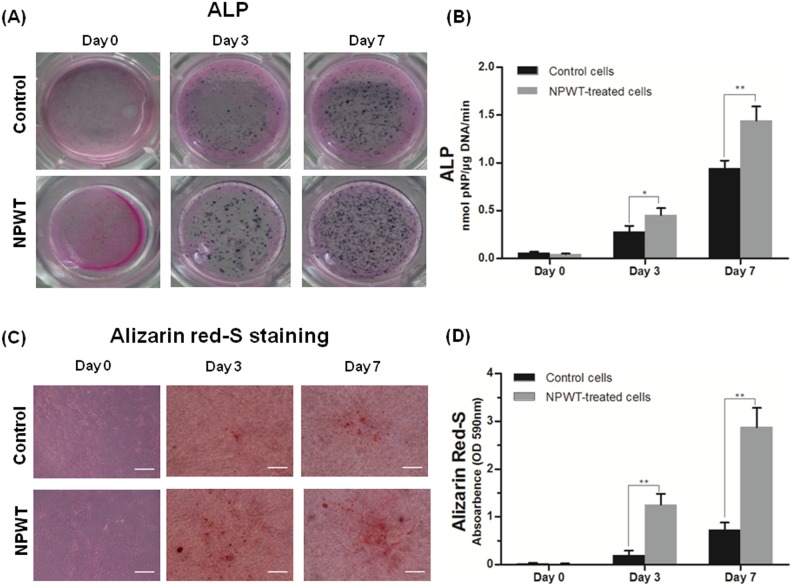
The expression of ALP and alizarin red-S staining. (A) Images of ALP staining of rat P-MSCs with or without NPWT treatment of different daily duration for 7 days. (B) ALP activity in P-MSCs treated with NPWT was notably higher than that in control group (*p<0.05, **p<0.01). (C) Result of alizarin red-S staining was in accordance with ALP staining (Bars = 50 µm). (D) Result of semi-quantitative analysis of alizarin red-S showed that absorbance index in NPWT group was significantly higher than that in control group (**p<0.01).

ALP staining showed that a deeper black-blue color could be seen in NPWT group than that in control group (Figure 4A). Alizarin Red staining showed that there were nearly no mineralization dots in control group, but sporadic, brownish red and opaque calcified nodules could be observed in the NPWT group after treated for 3 days. At day 7, the mineralization nodules were much bigger and denser in NPWT group (Figure 4C). Quantification assay showed significant increases in ALP activity and calcification in the NPWT group compared to the control (Figure 4B, D).

**Figure 5:**
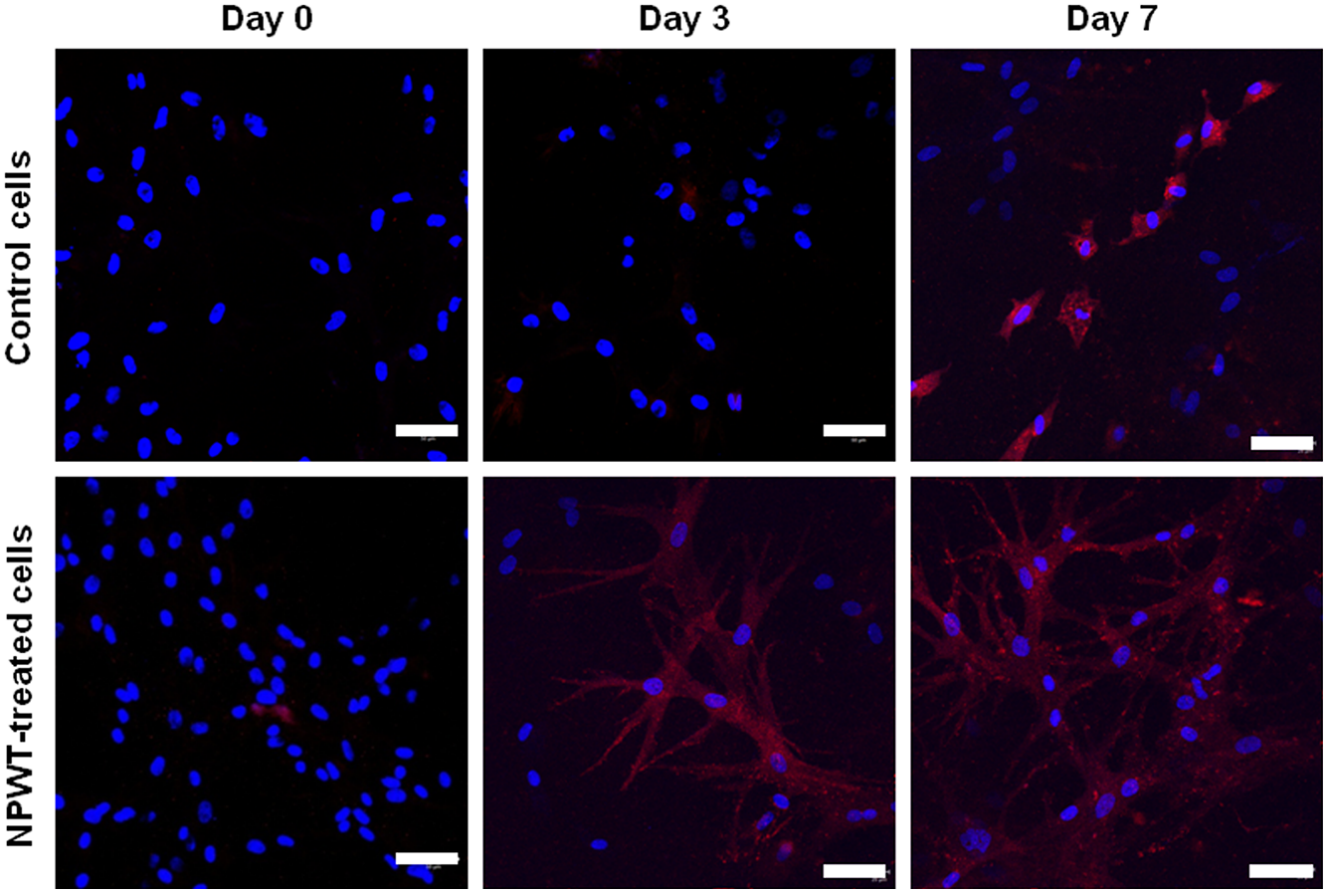
Immunofluorescence staining for COL1. Immediately before and 3, 7 days after incubation, cell clots were harvested and confocal microscopy was performed to detect fluorescent immunostaining for COL1. Results showed that the expression of COL1 was absent or weak in two groups before incubation, but stronger in NPWT group at day 3 and 7 after incubation. Bars = 50 µm.

Immunofluorescence staining showed that many cells in the clots incubated with NPWT for 3 days were shown to be stained positive for COL1, and the more so at day 7. However, very little stain was observed in the cells cultured without suction at day 3 and only a part of cells were positive at day 7 (Figure 5).

**Figure 6:**
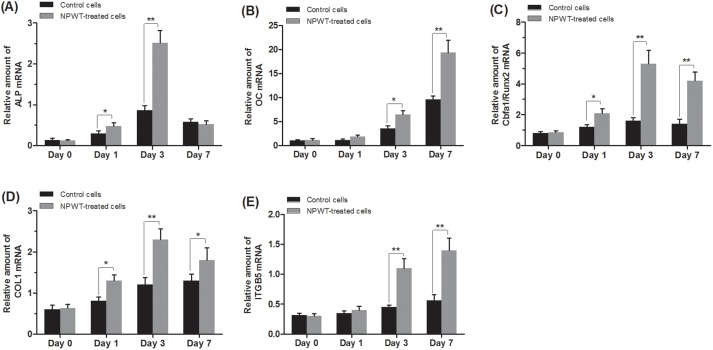
Real-time RT-PCR analysis. NPWT-treated and static control group cell clots were harvested before and 1, 3, and 7 days after incubation (n = 3 per timepoint for each group) and analyzed by real-time RT-PCR. The expressions of the specific osteogenic genes (A) ALP, (B) OC, and (D) COL1 were higher in NPWT group than those in control group (*p<0.05, **p<0.01). The NPWT group also expressed higher levels of the osteogenic transcription factor Cbfa1 (also known as Runx2) and the mechanosignaling molecule integrin β5 (ITGB5) (C) and (E) (*p<0.05, **p<0.01).

**Figure 7:**
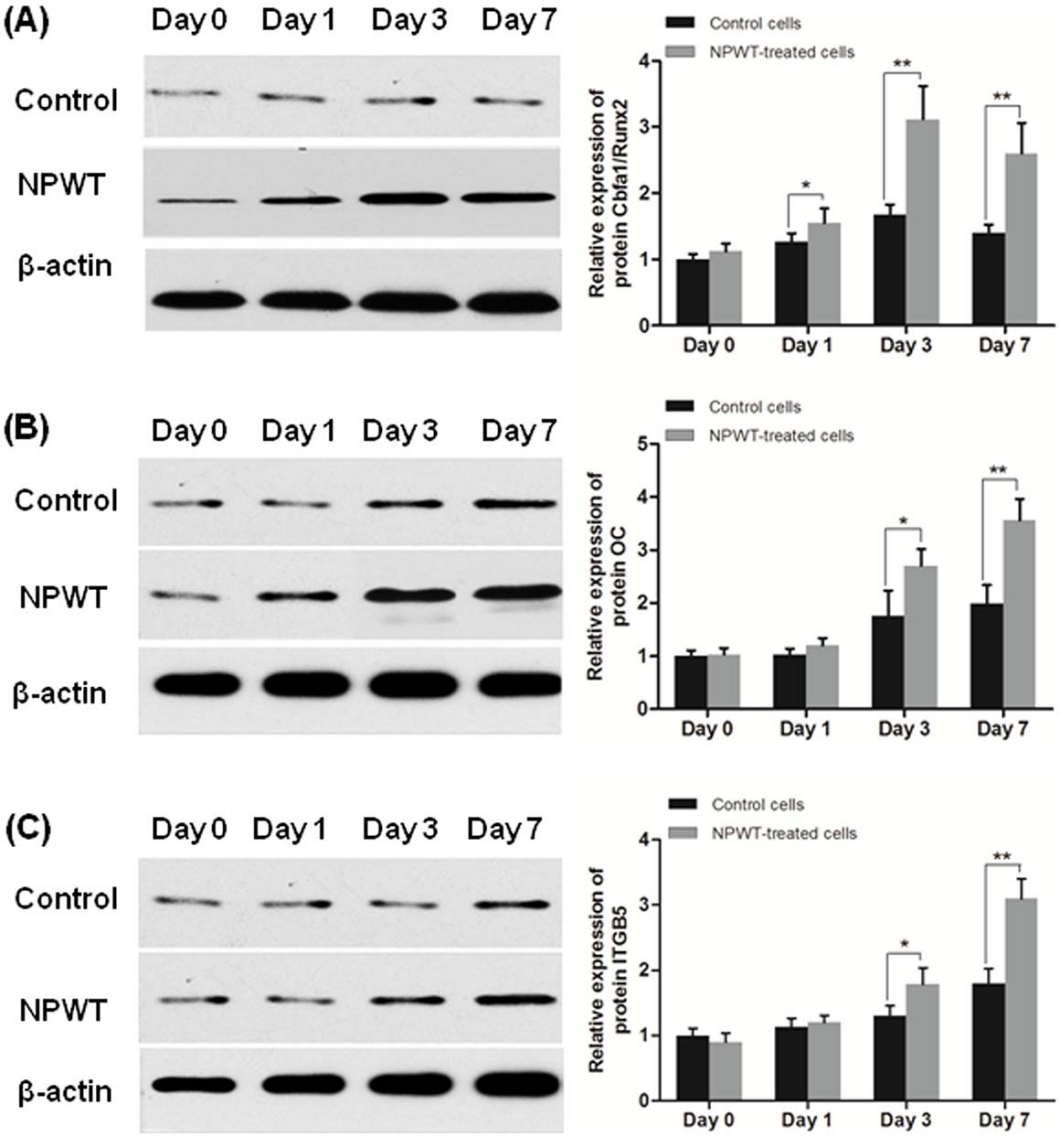
Western-blot assay. Protein expression levels of osteogenic factors in P-MSCs treated with NPWT at different time points were shown in (A) and (B). (A) Cbfa1/Runx2 was statistically overexpressed at day 1 after treatment with NPWT, with the increase being more significant after day 3. (B) Compared with control group, the expression of OC was markedly upregulated in NPWT group after day 3. ITGB5 protein level was shown in (C), it was not affected at day 1, but was strikingly upregulated after day 3 in NPWT group. (*p<0.05, **p<0.01).

In NPWT group, OC mRNA expression profiles showed an early increase at day 3, after that a significant upregulation at day 7 was detected. The mRNA expression of ALP, COL1 and Cbfa1/Runx2 peaked at day 3, and then slight decline was detected at day 7. The mRNA levels of ALP exhibited no significant differences among the two groups at day 7 (Figure 6). Western-blot analysis was used to confirm the expression changes of Cbfa1/Runx2 and OC, and the results were consistent with real-time RT-PCR (Figure 7A, B).

### ITGB5 mRNA and protein expression

Compared to the control group, the ITGB5 mRNA expression was significantly increased at day 3 and 7 (Figure 6E). Western-blot also showed that its protein expression was increasing slightly during 7 days treatment with NPWT (Figure 7C).

## Discussion

It’s well known that NPWT is a highly successful and widely used therapeutic method for wound healing. To date, many papers have been published on its action mechanism and application in various acute, chronic and complex wounds. However, little is known about its effect on bone healing. In this in vitro study, we provide new evidence that NPWT may play a positive role in the process of bone reconstruction and remodeling by strengthening the contribution of P-MSCs in fracture repair. This is supported by a) the enhanced proliferation of P-MSCs observed under NPWT treatment for 3 days; b) the significantly increased ALP activity and cell mineralization after treatment with NPWT for 3 and 7 days; c) the elevated expression of the osteoblastic-related markers at mRNA and protein levels after application of NPWT to P-MSCs.

As far as we know, this may be the first study about the effects of NPWT (continuous suction at −125 mmHg) on P-MSCs using a perfusion bioreactor which was designed by K.C.I. research group [22]. Many researchers have reported that fibroblasts treated with NPWT exhibit greater cell survival, migration, proliferation and levels of growth factors using this bioreactor [25], [26]. In our study, NPWT also increased the proliferation of P-MSCs at 72 h in continuous suction manner. There is no significant difference about cell apoptosis rate between two groups at this timepoint. However, this result may differ from previous reports [27], [28]. They showed that the proliferation of bone marrow-derived MSCs (B-MSCs) was inhibited and apoptosis was induced under low-intensity and intermittent negative pressure incubation. Except for the different pressure value, suction manner and laboratory environment, we hypothesize that other two reasons may contribute to these differences.

First, the bioreactor used in our experiment may be better for investigating the application of NPWT in vitro. As the report from K.C.I., we assembled multiple units of NPWT system and applied them on P-MSCs clots. Though the action mechanisms of NPWT are not fully understood, many studies have pointed that cell response is related to the polyurethane foam, whereas the tissue strain induced by the NPWT system stimulate cell proliferation [29]. McNulty et al [25] compared the viability, chemotactic signaling and proliferation of fibroblasts with different dressing, their results indicated that the dressing material has a significant effect on cell response following NPWT, the in vitro application of NPWT with foam support cell growth, proliferation and without increasing apoptosis. The mechanical stress created by application of NPWT principally thought to be via mechanical stretch and fluid-shear stress [30]. The effect of mechanical strain or hydrostatic pressure on the proliferation of osteoblastic cells is still controversial. In some studies, appropriate mechanical stress can increase proliferation of B-MSCs [16], while in others, the proliferation rate was reduced [31]. In our study, the cells in clots treated with NPWT may be stimulated by mechanical stretch and fluid-shear stress together. Mechanical stimuli through the suction of negative pressure source and hydrostatic pressure created by fluid media controlled by the peristaltic pump may conjointly cause the proliferation of P-MSCs in fibrin matrix.

Second, as we know, cell growth is related to cell shape [32]. Application of NPWT to P-MSCs induced the changes in their morphology. This change was consistent with the previous report [26]. How the cell shape control cell-cycle is unclear. Huang and Ingber [33] showed that changes in cell shape were related to the configuration of internal cell structures, including microfilaments and microtubules. For cells to respond to soluble mitogenic factors, they must extend and generate isometric tension by adherence to a stiff substrate or extracellular application of mechanical force. The cellular adhesion to ECM, cell shape and mechanical tension in cytoskeleton are very important for the local control of cell-cycle progression. In this study, P-MSCs were cultured in a three-dimensional fibrin matrix. So, we hypothesize that micromechanical deformation of the fibrin matrix induced by foam and suction results in extracellular matrix (ECM) distortion, then may change single cell shape and make the cellular proliferation response.

To further explore the events involved during osteogenic differentiation of P-MSCs under treatment with NPWT in vitro, characterization of the expression of several osteogenic markers was performed. ALP and Alizarin Red S staining denoted early differentiation and production of mineralized matrix nodules. The results showed that increased ALP expression and significant enhancement of mineralization in P-MSCs treated with NPWT compared to control cells. Cbfa1/Runx2 is a key transcription factor in osteoblastogenesis. Our data showed that production of Cbfa1/Runx2 initially increased but then decreased in response to a long period of NPWT treatment. This result was consistent with the report from Huang and Rei [17], and opposite to the reports about osteogenic response of MSCs to continuous mechanical strain [16], [34]. We hypothesize that both mechanical stretch and hydrostatic pressure contribute to the osteoblastic differentiation of P-MSCs, and the cells in fibrin matrix were more sensitive to fluid-shear stress, which may play a leading role in the mechanical stimuli created by NPWT. Detection of other osteogenic markers, such as COL1 and OC, also showed marked increase when P-MSCs were treated with NPWT.

Integrins are important mechanotransduction molecules that connect the major ECM components with the intracellular cytoskeleton and control ostoblasts differentiation and fate. Many studies reported the role of α5β1 and β1 subunits in osteoblasts differentiation and maturation. The integrin-ECM interactions could trigger many kinds of downstream signal pathways that converge to promote early osteoblast-specific gene expression in state with mechanical loading [35]. Huang et al [17] firstly reported that the β5 subunit of integrin was expressed in response to mechanical stimulation in MSCs. They suggested that integrin β5 may serve as a mechanotransducer in the osteogenic differentiation of MSCs promoted by hydrostatic pressure. In this study, we detected the expression of integrin β5 in two groups, and also found it expressed at a higher level after treatment with NPWT for 3 and 7 days. It has been proven that osteoclast maturation is accelerated in mice that lack integrin β5 subunit [36]. And, the expression level of integrin β5 in osteoclast from autosomal dominant osteopetrosis type II (ADO II) patients is higher than normal donors [37]. These results indicate that integrin β5 could inhibit osteoclast formation and may participate in NPWT driven oseogenesis through enhance osteoblastic differentiation and decrease osteoclast formation. This may be one possible mechanism for the promoted osteoblastic differentiation process of P-MSCs induced by NPWT in vitro.

We believe that the osteogenic induction of P-MSCs treated with NPWT is very complex. Many signals may participate in this process. The detailed mechanism studies are required to fully elucidate the mechanosignaling pathways at RNA and protein levels. It’s also essential to try other pressure values and time of treatment to find optimal conditions for promoting osteogenesis. Further work will be conducted using an animal model of fracture to confirm the role of NPWT in bone healing in vivo.

## Conclusion

In summary, we explored the influence of NPWT on P-MSCs proliferation and osteogenic differentiation in a 3D fibrin matrix. Our results show that a short time treatment with continuous suction at −125 mmHg could induce the differentiation of P-MSCs toward an osteogenic phenotype associated with enhancement of cellular proliferation. The mechanotransduction molecule integrin β5 was found to be highly expressed after NPWT treatment, which indicates that NPWT may promote fracture healing through enhancing bone formation and decreasing bone resorption. We hope this pilot study provided a scientific basis to prove the positive role of NPWT in bone healing and find the most beneficial waveforms of application in treatment of complex wound with fracture or bone defect.

## Supporting Information

Figure S1 **Characterization of P-MSCs.** **A**: Cell surface markers of P-MSCs (Passage 3) were analyzed by Fluorescence-activated cell sorting (FACS). P-MSCs expressed CD44 and CD90, but not CD34, CD45. **B**: Adipogenic differentiation was confirmed using Oil Red-O staining (×100). **C** and **D**: Osteogenic differentiation was revealed with ALP staining and alizarin red staining (×100).
(TIF)
