# Supplementary figures and images for: Effects of Negative Pressure Wound Therapy on Mesenchymal Stem Cells Proliferation and Osteogenic Differentiation in a Fibrin Matrix
Source: PLoS One. 2014 Sep 12;9(9):e107339. doi: 10.1371/journal.pone.0107339 (PMC4162584; doi:10.1371/journal.pone.0107339)

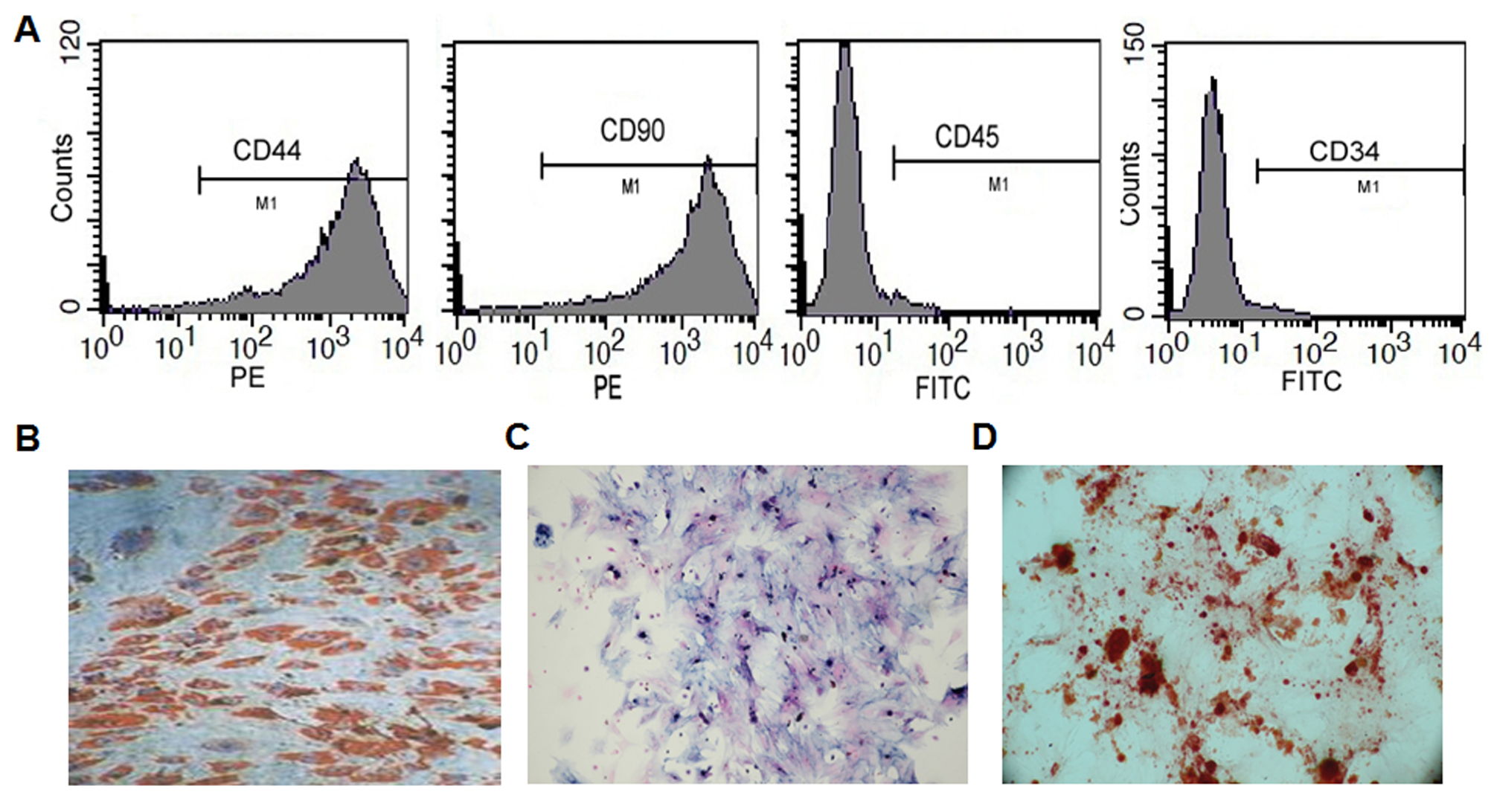

Supplement: Figure S1 — Characterization of P-MSCs. A: Cell surface markers of P-MSCs (Passage 3) were analyzed by Fluorescence-activated cell sorting (FACS). P-MSCs expressed CD44 and CD90, but not CD34, CD45. B: Adipogenic differentiation was confirmed using Oil Red-O staining (×100). C and D: Osteogenic differentiation was revealed with ALP staining and alizarin red staining (×100). (TIF) [file pone.0107339.s001.tif]
